# On the Consistency of the Exfoliation Free Energy of Graphenes by Molecular Simulations

**DOI:** 10.3390/ijms22158291

**Published:** 2021-08-02

**Authors:** Anastasios Gotzias, Elena Tocci, Andreas Sapalidis

**Affiliations:** 1 National Centre for Scientific Research “Demokritos”, Institute of Nanoscience and Nanotechnology INN, 15310 Athens, Greece; a.sapalidis@inn.demokritos.gr; 2 Institute on Membrane Technology ITM–CNR, National Research Council, 87036 Rende, Italy; e.tocci@itm.cnr.it

**Keywords:** liquid exfoliation, layered materials, decoupling simulations, umbrella sampling

## Abstract

Monolayer graphene is now produced at significant yields, by liquid phase exfoliation of graphites in solvents. This has increased the interest in molecular simulation studies to give new insights in the field. We use decoupling simulations to compute the exfoliation free energy of graphenes in a liquid environment. Starting from a bilayer graphene configuration, we decouple the Van der Waals interactions of a graphene monolayer in the presence of saline water. Then, we introduce the monolayer back into water by coupling its interactions with water molecules and ions. A different approach to compute the graphene exfoliation free energy is to use umbrella sampling. We apply umbrella sampling after pulling the graphene monolayer on the shear direction up to a distance from a bilayer. We show that the decoupling and umbrella methods give highly consistent free energy results for three bilayer graphene samples with different size. This strongly suggests that the systems in both methods remain closely in equilibrium as we move between the states before and after the exfoliation. Therefore, the amount of nonequilibrium work needed to peel the two layers apart is minimized efficiently.

## 1. Introduction

Graphenes are two-dimensional, single-layer carbon nanosheets with unprecedented physical, mechanical, optical and electronic properties [1,2,3,4]. They are classified with different layered materials like boron nitrides, metal oxides, dichalcogenides and the recently introduced class of metal organic framework nanosheets (MONs) [5,6,7]. Most of their fascinating properties are attributed to the atomic-scale thickness, the continuous 2D connectivity and the ultra-large specific surface area. Graphene nanosheets can be produced by liquid-phase exfoliation and dispersion of graphites [8,9,10,11,12]. This is achieved by chemical functionalization and sonication of graphitized matrices in the presence of certain solvents. Highly polar solvents can destroy the graphitic structure or leave defects and functional moieties on the dispersed layers. By definition, the defects and functional groups change the intrinsic atomic structure and the electrical and mechanical properties of graphene. Graphene oxide, for example, is an insulator rather than a semi metal, and therefore, it is conceptually different than graphene. The special properties of graphenes are also expected to change as the number of layers increases. This is important, because partial exfoliation may release multilayers and clusters instead of large scale of graphene monolayers. In addition, the exfoliated monolayers tend to aggregate into multilayer configurations within the solvent. Nowadays, several high-yield exfoliation methods have been reported that produce unoxidized and defect-free graphene. The material is used to produce graphene-based composites or films, a key requirement for applications such as thin-film transistors, conductive transparent electrodes, photovoltaics and biomedical implants [13,14,15]. The availability of high-quality graphene samples has increased the interest for explicit molecular simulations of the exfoliation processes and relevant applications [16,17,18,19].

The energy required to exfoliate graphene is balanced by the solvent-graphene interactions [9]. Solvent molecules enter the inner core of the graphite and break the Van der Waals forces between the layers. In order to simulate exfoliation, we configure paths either by pulling one layer at a distance from the remaining structure or by breaking the interactions of the layer in small changing steps [20,21]. If the steps are effectively small, the changes on the system are considered reversible, and the reversible work needed to transform one state into the other is equal to the free energy difference of the states before and after exfoliation. The free energy difference between two states, labeled A and B, is given by ΔF = $F_{A}-F_{B}$. ΔF is related to the ratio of the partition functions of the states [22]. If the states lie far apart in phase space, the estimation of this ratio is intractable [23,24]. The reversible path serves to overcome this hurdle. It provides a connection between the two states so that we can evaluate the changes in *F* using thermodynamic integration [25,26,27]. That is, we split the overall perturbation into small perturbation steps for which the phase spaces continuously overlap. In this respect, the ratio of the partition functions for the consecutive perturbation steps is calculated more easily [28,29].

We may configure various paths to transform state A into state B [30,31]. A common approach is to use a reaction coordinate to move reversibly from the A-like state to a B-like state. Another approach is to modify the system’s Hamiltonian. In this case, we mix the energies or the parameters of states A and B, according to a decoupling parameter, λ, which varies from 0 to 1 [32,33,34]. In many circumstances, it is convenient to introduce an intermediate state (or states) labeled C and evaluate the free energy difference by subtracting the free energies with respect to the state C: ΔF = $\left(F_{A}-F_{C}\right)-\left(F_{B}-F_{C}\right)$ [35,36,37,38,39].

In a previous work, we employed one method to compute the exfoliation free energy of graphenes [40]. In brief, we considered a normal and a shear reaction coordinate to exfoliate (dissociate) a graphene layer from a bilayer configuration in an aqueous solution. We computed the free energy differences using umbrella sampling in small steps along the exfoliation paths. We reported that the free energy difference was greater when the exfoliation was coordinated on the shear than the normal direction. This was attributed to the awareness that the shear exfoliation of graphenes is reversible whereas the normal exfoliation is not. Notably, this outcome is in accordance with experiment. For instance, ball milling utilizes high shear force to delaminate layered materials and generate 2D nanosheets [41,42]. In this regard, molecular simulations succeed to realize a proof of principle. That is, the slip between the layers takes place in the in-plane direction, under the effect of shear force to yield free-standing graphene monolayers.

In this work, we compute the exfoliation free energy of graphenes using decoupling molecular simulations. The simulations are performed in two stages. In the first stage, we decouple the Van der Waals interactions of a single layer starting from a bilayer graphene configuration, in the presence of saline water. In the second stage we decouple the interactions of the same layer starting from a single layer configuration. The exfoliation free energy is computed by subtracting the free energy differences between the two stages. We consider three bilayer graphene samples with small, medium and large size. Then, we compare the free energy estimates of the decoupling simulations with those of the umbrella sampling in which, the same graphene nanosheets dissociate on the shear direction in respect to the bilayer plane.

## 2. Materials and Methods

Solvation free energies were computed by simulation by decoupling a nanoparticle from the solvent by thermodynamic integration, using the idendity,

(1) $$\Delta F=\int_{0}^{1}d\lambda \langle \frac{\partial H(\lambda )}{\partial \lambda }\rangle$$

where *H* is parameterized Hamiltonian, and λ is the decoupling parameter, which parameterizes the atomistic interactions between the solvent and the nanoparticle [43,44]. The coupled state (λ=0) corresponds to a simulation where the nanoparticle is interacting fully with the solvent, whereas the uncoupled state (λ=1) corresponds to a simulation where the nanoparticle is not interacting with the solvent. Considering the van der Waals interaction between two atoms *i* and *j*, the λ-dependent Lennard Jones expression takes the form,

(2) $$U_{ij}={\left(1-\lambda \right)}^{n}4\epsilon_{ij}\left(\frac{1}{{\left[\alpha \lambda^{n}+{\left(\frac{r_{ij}}{\sigma_{ij}}\right)}^{6}\right]}^{2}}-\frac{1}{\alpha \lambda^{n}+{\left(\frac{r_{ij}}{\sigma_{ij}}\right)}^{6}}\right)$$

where $r_{ij}=\left|r_{i}-r_{j}\right|$ is the interatomic distance, $\epsilon_{ij}$ and $\sigma_{ij}$ are the Lorentz-Berthelot combination parameters. We set α=0.5 and n=1. The term, $a\lambda^{n}$, at the denominators makes the potential convergent as r→0, for λ<1. This is useful especially for advanced steps of the decoupling (0.7<λ<1) as it improves the sampling at distances close to $\sigma_{ij}$ from the atoms of the solute [32,45,46].

To configure the bilayer graphenes we considered two copies of a single layer graphene. The single layers were all-carbon sheets, having a honeycomb pattern of carbons and peripheral hydrogens. The layers were planar and square. We generated the coordinate files of the graphene layers using the BuildCstruct script (http://chembytes.wikidot.com/buildcstruct, accessed on 28 July 2021) [47]. We built three graphene layers with edges 1.0 nm, 1.5 nm and 2.5 nm. Using these structures, we configured three bilayer graphenes with small, medium and large size, respectively. We placed the copied layers in a parallel orientation, at a distance 0.3 nm from each other. We modeled the $sp^{2}$ carbon atoms on the basis of the OPLSAA references of naphthalene and of aliphatic carbons [48,49,50,51]. We modeled hydrogen interactions using the interaction parameters of benzene hydrogens. The structures were uncharged. We used the GROMACS package, version 2018 to build the simulations [52]. All simulations were submitted to the high performance computing services of the Greek National Infrastructure for research and technology, GRNET-ARIS.

We placed the bilayer graphene at the center of a cubic box. We set the nearest edge of the cube at 1.5 nm from the outermost atom of the bilayer. We set the cut off radius to 1.4 nm to be consistent with the minimum image convention. We used periodic boundary conditions in all directions. We solvated the simulation box with simple point charge (SPC) water and we added 100 mM NaCl. Ions were interacting fully with the SPC molecules. They were not interacting electrostatically with the bilayer, because the graphenes were uncharged. Complementary, we performed equivalent simulations for single layer graphene samples with small, medium and large size. We followed the same simulation protocol as for the bilayer graphenes, only that we used a single layer instead of two. The dimensions of the simulation cubes for all the studied systems and the number of molecules described therein, are listed in Table 1.

Before the decoupling simulations, the system energy was relaxed using a steepest descent minimization, followed by an equilibration in the NPT ensemble over 1 ns. We used the Berendsen coupling to regulate the temperature at 310 K and the pressure at 1 bar. The final configuration of the last NPT run, was used as starting configuration for the decoupling simulations. The bilayer graphene and the solvent (water and ions) were coupled to separate coupling paths. We used the Nose-Hoover method for the temperature coupling and the Parinello-Rahman for the pressure. We labeled the two graphene layers using a different index. One layer served as an immobile reference on which, we applied position restraints on the atoms. The other layer was interacting with the reference layer and the ambient molecules on the basis of Equation (2). We used 31 λ steps evenly distributed in [0,1], with dλ=0.032. Simulations for each of the 31 total λ steps ran over 10 nanoseconds. The thermodynamic integration was computed using the Bennett acceptance ratio (BAR) method [25].

We considered a two-stage process to compute the exfoliation free energy of graphenes. First we deleted a single graphene sheet from the bilayer configuration, then we solvated the graphene sheet back into the solvent. The free energy difference on the first stage was computed by the decoupling simulations of bilayer graphene samples. Likewise, the free energy difference on the second stage was computed by the decoupling simulations of single layer graphenes. The exfoliation free energy was estimated by subtracting the free energy difference on the second stage from that on the first stage.

A different technique to compute the exfoliation free energy of bilayer graphenes is to use umbrella sampling simulations. The umbrella sampling method was discussed in detail in a previous work [40]. There, we employed umbrella sampling to compute the binding free energies of the same, as in this work, three bilayer graphene samples. Although we used the term “binding” instead of “exfoliation” in the definition of the free energy, we refer to the same thermodynamic quantity. In brief, we configured a path in which a single graphene layer dissociated from a bilayer configuration (state A) to an arbitrary far distance (state B) in the solvent. This was achieved, by setting a force to pull the graphene along the coordinate of the path. The other layer (reference) remained at a fixed position. We performed umbrella sampling on a sequence of configuration points along the dissociation path. Each umbrella simulation output a probability distribution function. It was critical, that the neighboring umbrella distributions along the sequence of configurations were overlapping. If two consecutive umbrella distributions did not overlap, we sampled more configuration points until the new distributions bridged the non-overlapping gaps. In this respect, the spacings between the sampled configurations on the path from state A to state B, could be uneven or arbitrarily small. After completing a sufficient amount of umbrella sampling, we gathered the corresponding probability distributions and computed the free energies using the weighted histogram analysis method (WHAM) [53].

## 3. Results

**Figure 1:**
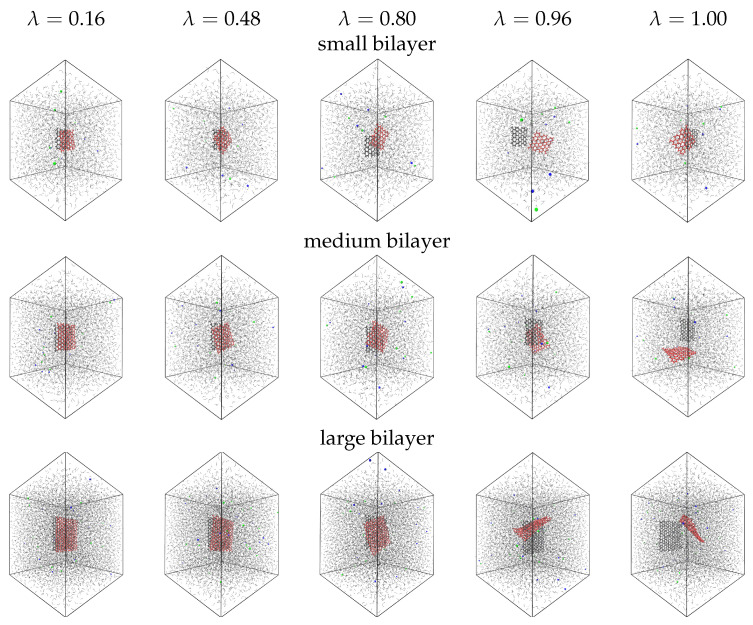
Configurations of the decoupling simulations of small, medium and large bilayer graphene samples at different values of the decoupling parameter, λ. As λ increases, the van der Waals interactions of the decoupled graphene layer decrease. The decoupled graphene layer is colored in red and the reference in black. Water molecules are shown with lines. Na${}^{+}$ and Cl${}^{-}$ ions are shown with red and blue spheres. The time-frame of the presented configurations is random.

In Figure 1, we present configurations from the decoupling simulations performed on three bilayer graphene samples with small, medium and large size. We place the samples at the centre of a cubic box and solvate with saline water. In Figure 1, we decouple only the red graphene layer. The black layer interacts regularly with the environment and serves as a reference. We scale down the Van der Waals interactions of the decoupled graphene by increasing the parameter λ on the basis of Equation (2). At small λ values, the bilayer configurations are stable due to the adequately strong interlayer interactions. The layers preserve a parallel orientation and they can only twist at small angles against each other. With increasing λ, the Van der Waals interactions of the decoupled graphene decrease. This makes the decoupled graphene disconnect from the reference layer. As λ → 1, the decoupled graphene moves freely inside the box. The decoupling interactions become small enough, so that we may to observe configurations in which the two layers intersect. This is due to the term $\alpha \lambda^{n}$ on the denominators of Equation (2), which makes the Van der Waals potential go to zero in a well-behaved manner with λ. The reference layer remains at a fixed position through the simulations, by imposing position restraints on the atoms. However, we can see in the snapshots of Figure 1 that when the two layers are apart, the reference layer is displaced from the box centre to the opposite direction of the decoupled graphene. This is because we change the periodicity on the representation of the simulated trajectories, setting the center of mass (COM) of both layers at the center of the simulation box.

**Figure 2:**
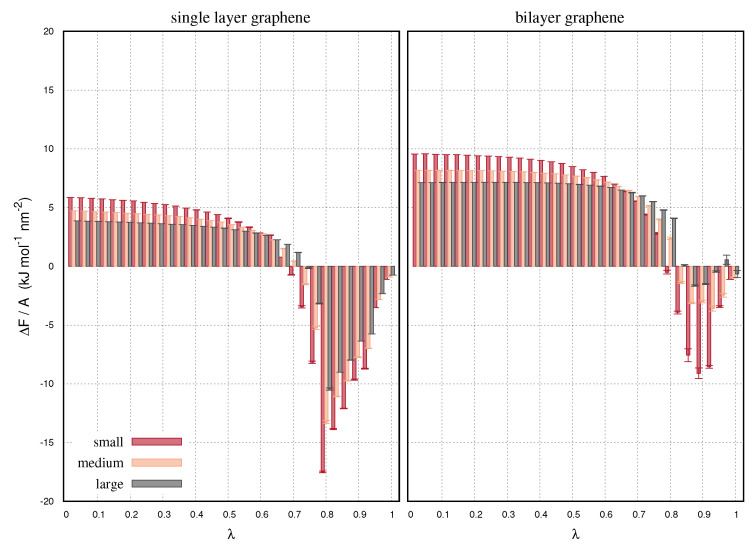
Free energy derivatives as a function of the decoupling parameter, λ, for the single and bilayer graphene samples with small, medium and large size. The free energy values are normalized by the area size of the graphenes.

Figure 2, shows the derivatives of the free energy as a function of the decoupling parameter, λ. Increasing λ from zero to one represents a gradual change from a system with full Van der Waals interactions, to a system where the decoupled graphene is not interacting with the environment. We use 31 λ steps evenly distributed in [0,1]. We consider both single layer and bilayer configurations. In the bilayer configurations only one layer is being decoupled. The free energy values are normalized by the area of the graphenes in order to highlight the finite-size effects of the calculations. Such effects are expected to be appreciable for small interfaces. As a result, small graphenes obtain greater free energy derivatives than the large. We also observe greater energy derivatives for the bilayer than the single layer configurations. This is due to the additional interactions of the second layer. At the low λ range, the energy difference is positive indicating the stability of the starting configurations. At advanced steps of the decoupling, λ > 0.7, the free energy difference is negative. At high λ range, the decoupling interactions are near zero so that the water molecules are allowed to sample the volumes occupied by the atomic sites of the decoupled graphene. This is the same reason why we may observe the layer intersections in Figure 1. The small interaction energy implies a rearrangement of the solvent phase that creates new, lower in energy configurations, making the energy decrease. The bilayer graphenes obtain a first negative energy difference at higher λ, than the single layer graphenes. This is because of the interactions of the reference layer that do not change upon decoupling. Likewise, the negative energy differences of the bilayers are smaller than those of the single layer configurations.

**Figure 3:**
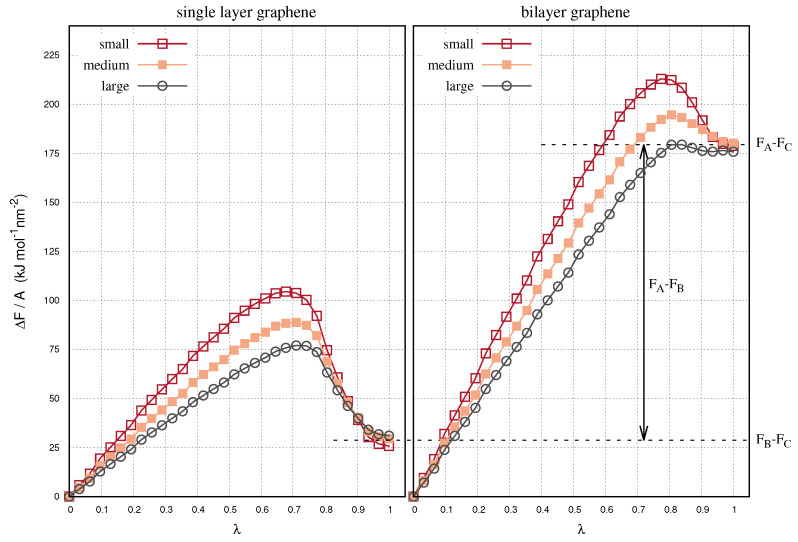
Cumulative free energies as a function of the decoupling parameter, λ, for the single and bilayer graphene samples with small, medium and large size. The free energy values are normalized by the area size of the graphenes.

In Figure 3 we show the relative free energies of the decoupling simulations as a function of λ. The energies result from the summation of the energy derivatives shown in Figure 2, up to the value of λ. Using decoupling simulations we simulate the transformation of a single layer (state B) or of a bilayer (state A) into a system where one layer is removed (state C). At full decoupling (i.e., λ=1) the relative free energy of the single and the bilayer graphenes is expressed by $F_{B}-F_{C}$ and $F_{A}-F_{C}$, respectively. In the panels of Figure 3, the free energies converge nearly on the same value, regardless of the size of the layers. This is important as it approves that we used appropriate spacings dλ in the thermodynamic integration. The convergence of the free energy differences also confirms the statistical consistency of our calculations, since the contributing integrals have resulted from individual simulations.

**Figure 4:**
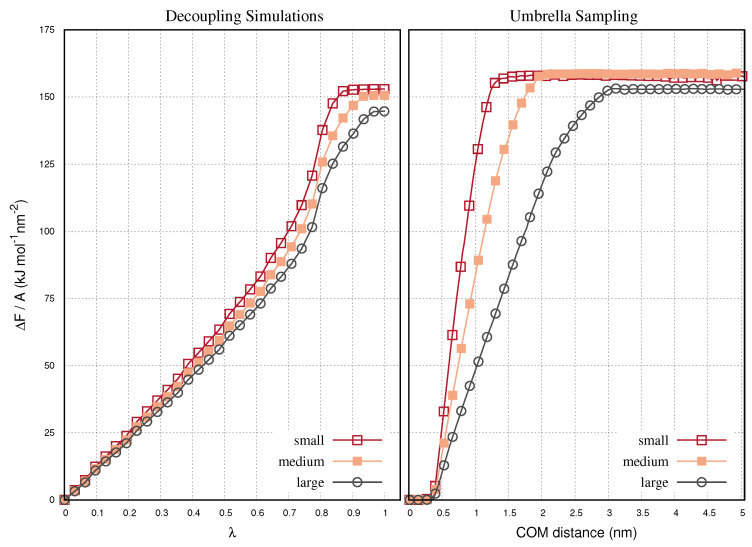
(**Left**) Exfoliation free energies of bilayer graphene samples with small, medium and large size, computed by decoupling simulations. The energies are shown as a function of the decoupling parameter λ, and computed by subtracting the relevant curves shown in the panels of Figure 3, based on the expression $\Delta F_{AB}$ = $\left(F_{B}-F_{C}\right)$ − $\left(F_{C}-F_{A}\right)$. (**Right**) Exfoliation free energy of the same bilayer graphene samples computed by umbrella sampling. The energies are shown as a function of the interlayer distance as the one layer is pulled away from the other on the shear direction. The umbrella sampling simulations are performed in a previous work [40].

The exfoliation free energy of the bilayer graphenes is given by the difference between the relative energies in the two panels in Figure 3, i.e., $F_{AB}$ = $\left(F_{A}-F_{C}\right)$ − $\left(F_{B}-F_{C}\right)$. The free energies are plotted in the left-hand panel in Figure 4, as a function of the decoupling parameter, λ. The free energy is proportional to λ up to a range, where the energy presents a step-like increase. This step is attributed to the peak of the bilayer energy curves at 0.7 < λ < 0.9, in Figure 3. At higher values of λ, the energy values reach a plateau. At λ=1 we compute comparable exfoliation free energies for the three systems. In general, it is difficult to obtain converged free energy estimates by subtracting the energies of different Hamiltonians. The descrepancies of the free energy are attributed to the different contributions of the solvent-solvent interactions to the overall energy. Solvent-solvent interactions can be negligible compared to the magnitude of the solvent-graphene or the graphene-graphene interactions. However, when the graphene layers are small, the contributions of the solvent-solvent interactions may affect the result. The exfoliation free energies along with the values and statistical variances of $F_{B}-F_{C}$ and $F_{A}-F_{C}$ for the single and bilayer configurations are listed in Table 2.

For the sake of comparison, we plot the exfoliation free energies of the bilayer graphenes computed with umbrella sampling simulations, in the right-hand panel of Figure 4. The umbrella simulations are detailed in a previous work [40]. The exfoliated graphene is pulled on the shear direction relevant to the initial plane of the bilayer. It is pulled over 0.5 ns using a pull rate 10 nm ns${}^{-1}$, so that a final COM distance of 5 nm between the layers is achieved. At this distance, the layers do not interact with each other, so that we may assume the layer exfoliated. The free energies are expressed in Figure 4 as a function of the center of mass (COM) distance between the layers. The free energies obtain a plateau when the interlayer distance becomes greater than the lateral size of the graphenes. Pulling further the graphene, does not change the free energy of the system. The umbrella simulations give comparable free energies with the decoupling simulations for the same graphene samples. This means that the two methods, i.e., shear pulling umbrella sampling and decoupling of van der Waals interactions of the graphene layers, are similar in terms of reversibility.

## 4. Discussion

Free energy calculations are independent of the simulation protocol that is used. However, the level of precision does depend very much on the choice of the transformation path [54,55]. Different configurations having an absolute free energy $F_{A}$ should on application of an adiabatic transformation all end up with the same free energy $F_{B}$. If this condition is not satisfied the transformation has been carried out too rapidly and the transformation strictly speaking is not adiabatic. The states, A and B, before and after exfoliation are separated by a free energy barrier. With decoupling simulations, we cross the barrier by introducing an intermediate state C. With umbrella simulations, we cross the barrier with more umbrella sampling. Nevertheless, there are two distinct types of barriers. The first type is is due to interactions of the perturbed degrees of freedom i.e., those of the exfoliated layer with itself and the environment. The other type of energy barrier is attributed to the interactions of the non perturbed degrees of freedom, i.e., those of the solvent (water and ions). These interactions cannot be smoothed by adding intermediate states or with additional sampling. In this respect, the rearrangement of the solvent molecules can increase the system’s entropy affecting the free energy calculations.

Apart from the solvent phase perturbations, discrepancies of the free energy are often attributed to the statistical variance of the molecular simulations. We can see in Table 2 that the variances are small, however, they do not reflect the level of accuracy of the estimates. The error of a free energy computed by molecular simulation, $\Delta F_{sim}$, in respect to that of an experiment, $\Delta F_{exp}$, is given by

(3) $$\Delta F_{exp}=\Delta F_{sim}-kTln\left(V_{\lambda =1}/V_{\lambda =0}\right)$$

This equation involves only the average volume $V_{\lambda =0}$ of the fully coupled system, and average volume $V_{\lambda =1}$ of the box of pure solvent (or of the solvent and the reference layer) containing the same number of solvent molecules as the coupled system [32,56]. The term with the ratio of the volumes, i.e., the correction, for the single and bilayer decoupling simulations, is tabulated in Table 2. In each case, the correction is between 10% and 40% of the reported variance. Because of this magnitude we can safely neglect this term in our calculations.

## 5. Conclusions

The availability of high-yield exfoliation methods to produce isolated, unoxidized and defect-free graphene has increased the interest in fundamental studies on the exfoliation processes by molecular simulations. We compute the liquid-phase exfoliation free energy of three graphene layers with different sizes using two simulation protocols, namely decoupling molecular dynamics and umbrella sampling. Using the two simulations, we estimate very similar free energies regardless of the size of the layers. This reflects the thermodynamic consistency of the methodologies and confirms that we designed the corresponding exfoliation paths to be equally reversible. The modeled systems, containing graphenes and water and ions, are reasonably simple, to be able to obtain good statistical sampling, allowing us to realize a lower bound on the amount of the sampling necessary to simulate more complex structures like functionalized graphenes and other 2D layered materials and metal organic nanosheets.

## Figures and Tables

**Table 1:** Model properties of graphene layers with small, medium and large size, edge lengths of the simulation cubes and the number of solvent molecules (water and ions) contained in the cubes for the single and bilayer graphene configurations.

Graphene layer	Small		Medium		Large	
Side Length (nm)	1.0		1.5		2.5	
Carbon atoms	59		111		263	
Hydrogen atoms	23		31		47	
	**Single Layer Graphene**			**Bilayer Graphene**		
**Simulation Cube**	**Small**	**Medium**	**Large**	**Small**	**Medium**	**Large**
Cube edge (nm)	4.72	5.36	6.67	4.74	5.38	6.68
Water molecules (SPC)	3369	4929	9651	3411	4924	9608
Ions Na${}^{+}$, Cl${}^{-}$	6	9	18	6	9	18

**Table 2:** Differences of free energy by decoupling bilayer, $F_{A}-F_{C}$, and single layer, $F_{B}-F_{C}$, graphene samples with small, medium and large size. The free energy variance (±σ) is given by the average of the variances over the 31 λ steps. Correction is computed by $kTln(V_{\lambda =1}/V_{\lambda =0})$. $F_{AB}$ is the exfoliation free energy expressed by $\left(F_{A}-F_{C}\right)-\left(F_{B}-F_{C}\right)$. $F_{AB}^{u}$ is the exfoliation free energy for the corresponding bilayer graphene samples computed using umbrella sampling in a previous work [40]. The energies are normalized by the area of the graphene layers, expressed in kJ mol${}^{-1}$ nm${}^{-2}$.

	Bilayer Graphene		Single Layer Graphene			
	$\Delta F_{\mathrm{sim}}=F_{A}-F_{C}$	Correction	$\Delta F_{\mathrm{sim}}=F_{B}-F_{C}$	Correction	$F_{\mathrm{AB}}$	$F_{\mathrm{AB}}^{u}$
small	178.71 ± $5.28\times 10^{-2}$	$1.93\times 10^{-2}$	25.77 ± $1.84\times 10^{-2}$	$1.77\times 10^{-2}$	152.94	157.41
medium	180.12 ± $3.02\times 10^{-2}$	$8.76\times 10^{-3}$	29.55 ± $1.56\times 10^{-2}$	$8.89\times 10^{-3}$	150.57	158.59
large	175.73 ± $3.29\times 10^{-2}$	$3.89\times 10^{-3}$	31.08 ± $8.70\times 10^{-3}$	$4.00\times 10^{-3}$	144.65	152.88

## Data Availability

Upon request.

## References

[1] Backes C., Abdelkader A., Alonso C., Andrieux-Ledier A., Arenal R., Azpeitia J., Balakrishnan N., Banszerus L., Barjon J., Bartali R. (2020). Production and processing of graphene and related materials. 2D Mater..

[2] Sekhon S.S., Kaur P., Kim Y.H., Sekhon S.S. (2021). 2D graphene oxide–aptamer conjugate materials for cancer diagnosis. NPJ 2D Mater. Appl..

[3] SI A., Kyzas G.Z., Pal K., de Souza F.G. (2021). Graphene functionalized hybrid nanomaterials for industrial-scale applications: A systematic review. J. Mol. Struct..

[4] Pereira L.F.C. (2021). Investigating mechanical properties and thermal conductivity of 2D carbon-based materials by computational experiments. Comput. Mater. Sci..

[5] Sarkar A.S., Stratakis E. (2021). Dispersion behaviour of two dimensional monochalcogenides. J. Colloid Interface Sci..

[6] Zhang C.J. (2021). Interfacial assembly of two-dimensional MXenes. J. Energy Chem..

[7] Ashworth D.J., Foster J.A. (2018). Metal–organic framework nanosheets (MONs): A new dimension in materials chemistry. J. Mater. Chem. A.

[8] Hernandez Y., Nicolosi V., Lotya M., Blighe F.M., Sun Z., De S., McGovern I.T., Holland B., Byrne M., Gun’Ko Y.K. (2008). High-yield production of graphene by liquid-phase exfoliation of graphite. Nat. Nanotechnol..

[9] Coleman J.N., Lotya M., O’Neill A., Bergin S.D., King P.J., Khan U., Young K., Gaucher A., De S., Smith R.J. (2011). Two-Dimensional Nanosheets Produced by Liquid Exfoliation of Layered Materials. Science.

[10] Cui X., Shi W., Lu C. (2021). Large-scale visualization of the dispersion of liquid-exfoliated two-dimensional nanosheets. Chem. Commun..

[11] Vacacela Gomez C., Guevara M., Tene T., Villamagua L., Usca G.T., Maldonado F., Tapia C., Cataldo A., Bellucci S., Caputi L.S. (2021). The liquid exfoliation of graphene in polar solvents. Appl. Surf. Sci..

[12] Chen X., Dubois M., Radescu S., Rawal A., Zhao C. (2021). Liquid-phase exfoliation of F-diamane-like nanosheets. Carbon.

[13] Natter N., Kostoglou N., Koczwara C., Tampaxis C., Steriotis T., Gupta R., Paris O., Rebholz C., Mitterer C. (2019). Plasma-Derived Graphene-Based Materials for Water Purification and Energy Storage. C.

[14] Naeem M., Kuan H.C., Michelmore A., Yu S., Mouritz A.P., Chelliah S.S., Ma J. (2021). Epoxy/graphene nanocomposites prepared by in-situ microwaving. Carbon.

[15] Gotzias A. Injecting Carbon Nanostructures in Living Cells. Proceedings of the Workshops of the 11th EETN Conference on Artificial Intelligence 2020 (SETN2020 Workshops).

[16] Kong X., Zhuang J., Zhu L., Ding F. (2021). The complementary graphene growth and etching revealed by large-scale kinetic Monte Carlo simulation. NPJ Comput. Mater..

[17] Stevens K., Tran-Duc T., Thamwattana N., Hill J.M. (2020). Modeling Interactions between Graphene and Heterogeneous Molecules. Computation.

[18] Folorunso O., Hamam Y., Sadiku R., Sinha Ray S., Adekoya G. (2021). Comparative study of graphene-polypyrrole and borophene-polypyrrole composites: Molecular dynamics modeling approach. Eng. Solid Mech..

[19] Parab A.D., Budi A., Slocik J.M., Rao R., Naik R.R., Walsh T.R., Knecht M.R. (2020). Molecular-Level Insights into Biologically Driven Graphite Exfoliation for the Generation of Graphene in Aqueous Media. J. Phys. Chem. C.

[20] Liang L., Chen E.Y., Shen J.W., Wang Q. (2016). Molecular modelling of translocation of biomolecules in carbon nanotubes: Method, mechanism and application. Mol. Simul..

[21] Cai L., Lv W., Zhu H., Xu Q. (2016). Molecular dynamics simulation on adsorption of pyrene-polyethylene onto ultrathin single-walled carbon nanotube. Phys. E Low-Dimens. Syst. Nanostruct..

[22] Han K.K. (1992). A new Monte Carlo method for estimating free energy and chemical potential. Phys. Lett. A.

[23] Christ C.D., van Gunsteren W.F. (2007). Enveloping distribution sampling: A method to calculate free energy differences from a single simulation. J. Chem. Phys..

[24] Wu D., Kofke D.A. (2005). Phase-space overlap measures. II. Design and implementation of staging methods for free-energy calculations. J. Chem. Phys..

[25] Bennett C.H. (1976). Efficient estimation of free energy differences from Monte Carlo data. J. Comput. Phys..

[26] Frenkel D., Smit B. (1996). Understanding Molecular Simulation: From Algorithms to Applications.

[27] Schultz A.J., Kofke D.A. (2021). Identifying and estimating bias in overlap-sampling free-energy calculations. Mol. Simul..

[28] Wu D. (2010). Understanding free-energy perturbation calculations through a model of harmonic oscillators: Theory and implications to improve the sampling efficiency by molecular simulation. J. Chem. Phys..

[29] Sidler D., Schwaninger A., Riniker S. (2016). Replica exchange enveloping distribution sampling (RE-EDS): A robust method to estimate multiple free-energy differences from a single simulation. J. Chem. Phys..

[30] Perthold J.W., Petrov D., Oostenbrink C. (2020). Toward Automated Free Energy Calculation with Accelerated Enveloping Distribution Sampling (A-EDS). J. Chem. Inf. Model..

[31] Wu J.Z., Azimi S., Khuttan S., Deng N., Gallicchio E. (2021). Alchemical Transfer Approach to Absolute Binding Free Energy Estimation. J. Chem. Theory Comput..

[32] Shirts M.R., Pitera J.W., Swope W.C., Pande V.S. (2003). Extremely precise free energy calculations of amino acid side chain analogs: Comparison of common molecular mechanics force fields for proteins. J. Chem. Phys..

[33] Mobley D.L., Chodera J.D., Dill K.A. (2006). On the use of orientational restraints and symmetry corrections in alchemical free energy calculations. J. Chem. Phys..

[34] Fathizadeh A., Elber R. (2018). A mixed alchemical and equilibrium dynamics to simulate heterogeneous dense fluids: Illustrations for Lennard-Jones mixtures and phospholipid membranes. J. Chem. Phys..

[35] Wu D., Kofke D.A. (2005). Phase-space overlap measures. I. Fail-safe bias detection in free energies calculated by molecular simulation. J. Chem. Phys..

[36] Cournia Z., Allen B., Sherman W. (2017). Relative Binding Free Energy Calculations in Drug Discovery: Recent Advances and Practical Considerations. J. Chem. Inf. Model..

[37] Heinzelmann G., Gilson M.K. (2021). Automation of absolute protein-ligand binding free energy calculations for docking refinement and compound evaluation. Sci. Rep..

[38] Lee T.S., Allen B.K., Giese T.J., Guo Z., Li P., Lin C., McGee T.D., Pearlman D.A., Radak B.K., Tao Y. (2020). Alchemical Binding Free Energy Calculations in AMBER20: Advances and Best Practices for Drug Discovery. J. Chem. Inf. Model..

[39] Hahn D.F., Hünenberger P.H. (2019). Alchemical Free-Energy Calculations by Multiple-Replica *λ*-Dynamics: The Conveyor Belt Thermodynamic Integration Scheme. J. Chem. Theory Comput..

[40] Gotzias A. (2021). Binding Free Energy Calculations of Bilayer Graphenes Using Molecular Dynamics. J. Chem. Inf. Model..

[41] Ranjan R., Bajpai V. (2021). Graphene-based metal matrix nanocomposites: Recent development and challenges. J. Compos. Mater..

[42] Zhou D., Zhao L., Li B. (2021). Recent progress in solution assembly of 2D materials for wearable energy storage applications. J. Energy Chem..

[43] Noroozi J., Ghotbi C., Sardroodi J.J., Karimi-Sabet J., Robert M.A. (2016). Solvation free energy and solubility of acetaminophen and ibuprofen in supercritical carbon dioxide: Impact of the solvent model. J. Supercrit. Fluids.

[44] Bux K., Moin S.T. (2020). Solvation of cholesterol in different solvents: A molecular dynamics simulation study. Phys. Chem. Chem. Phys..

[45] König G., Glaser N., Schroeder B., Kubincová A., Hünenberger P.H., Riniker S. (2020). An Alternative to Conventional *λ*-Intermediate States in Alchemical Free Energy Calculations: *λ*-Enveloping Distribution Sampling. J. Chem. Inf. Model..

[46] Mecklenfeld A., Raabe G. (2017). Efficient solvation free energy simulations: Impact of soft-core potential and a new adaptive *λ*-spacing method. Mol. Phys..

[47] Shkolin A.V., Fomkin A.A., Yakovlev V.Y., Men’shchikov I.E. (2018). Model Nanoporous Supramolecular Structures Based on Carbon Nanotubes and Hydrocarbons for Methane and Hydrogen Adsorption. Colloid J..

[48] Jorgensen W.L., Maxwell D.S., Tirado-Rives J. (1996). Development and Testing of the OPLS All-Atom Force Field on Conformational Energetics and Properties of Organic Liquids. J. Am. Chem. Soc..

[49] Karataraki G., Sapalidis A., Tocci E., Gotzias A. (2019). Molecular Dynamics of Water Embedded Carbon Nanocones: Surface Waves Observation. Computation.

[50] Gotzias A., Sapalidis A. (2020). Pulling Simulations and Hydrogen Sorption Modelling on Carbon Nanotube Bundles. C.

[51] Tieleman D.P., MacCallum J.L., Ash W.L., Kandt C., Xu Z., Monticelli L. (2006). Membrane protein simulations with a united-atom lipid and all-atom protein model: Lipid–protein interactions, side chain transfer free energies and model proteins. J. Phys. Condens. Matter.

[52] Abraham M.J., Murtola T., Schulz R., Páll S., Smith J.C., Hess B., Lindahl E. (2015). GROMACS: High performance molecular simulations through multi-level parallelism from laptops to supercomputers. SoftwareX.

[53] Kumar S., Rosenberg J.M., Bouzida D., Swendsen R.H., Kollman P.A. (1992). The weighted histogram analysis method for free-energy calculations on biomolecules. I. The method. J. Comput. Chem..

[54] Lemkul J.A., Allen W.J., Bevan D.R. (2010). Practical Considerations for Building GROMOS-Compatible Small-Molecule Topologies. J. Chem. Inf. Model..

[55] Chelli R., Gellini C., Pietraperzia G., Giovannelli E., Cardini G. (2013). Path-breaking schemes for nonequilibrium free energy calculations. J. Chem. Phys..

[56] Hinkle K.R., Phelan F.R. (2017). Solvation of Carbon Nanoparticles in Water/Alcohol Mixtures: Using Molecular Simulation To Probe Energetics, Structure, and Dynamics. J. Phys. Chem. C.

